# Transactions of Iowa Med. Society

**Published:** 1856-08

**Authors:** 


### TRANSACTIONS OF THE IOWA STATE MEDICAL SOCIETY.

### SEVENTH SESSION—OTTUMWA.

JUNE 11, 1856, 2, P. M.

The Society was called to order by the President, Dr. Siveter.

In the absence of the Recording Secretary, Dr. Rauch of Burlington was, on motion of Prof. M'Gugin, appointed Secretary, *pro. tem.*

The Constitution and By-Laws were read and the proceedings of the last annual meeting.

The Censors being absent, on motion, Prof. Hughes, Dr. Cousins and Prof. M'Gugin were appointed Censors, *pro. tem.*

The board of Censors recommended the following named gentlemen as worthy of membership: Drs. A. D. Wood, Eli A. Harper, C. C. Warden, Jas. L. Taylor, J. Williamson, S. B. Thrall, Hawkins, A. Heuvell, and J. J. Douglass, Ottumwa; Wells R. Marsh and D. C. Dewey, Keokuk; A. C. Olney, Chillicothe; V. V. Adamson and J. W. Davidson, Nevada: I. I. Ellison, Wapello: J. M. M. Overholtz and J. W. LaForce, Ashland; D. Y. Collins and Chas. Fitch, Chariton; W. Gutch, Blakesburg; S. S. Norris, E. D. Black and A. R. Nim, Agency City; J. C. Johnson, Dahlonega.

The above named, on complying with the requirements of the Constitution and By-Laws, became members of the Society.

On motion, the order of business of the American Medical Association was adopted.

On motion, the President appointed Drs. Wood, Marsh, La Force, Fitch and Cousins a committee to nominate officers of the Society for the ensuing year. The committee reported as follows:

President, Prof. J. C. Hughes, Keokuk; Vice President, Dr. A. D. Wood, Ottumwa; Rec. Secretary, Dr. M. Cousins, Albia; Cor. Secretary, Prof. D. L. M'Gugin, Keokuk; Treasurer, Dr. J. Siveter, Salem; Librarian, Dr. Carpenter, Cedar Rapids.

*Censors.*—Dr. Wm. Gutch, Blakesburg; Dr. Reeder, Muscatine; Dr. Witherwax, Davenport; Dr. D. Y. Collins, Chariton; Dr. Henry, Burlington; Dr. V. Norris, Birmingham; Dr. V. V. Adamson, Nevada.

On motion, the article of the Constitution, requiring balloting for officers, was suspended.

The report was received, and the gentlemen named were unanimously elected officers of the Society for the ensuing year.

On motion of Professor McGugin, the retiring President delivered his valedictory address.

On motion of Dr. Rauch, it was resolved that the address be published, and that the thanks of the Society be tendered to the retiring President for the able and faithful manner in which he has discharged the duties of his office.

The President, Prof. Hughes, was then conducted to the Chair by Drs. Wood and Rauch.

The report of the former Secretary was read, when, upon motion, it was resolved that a committee of three be appointed to revise the Constitution and By-Laws, and report at the morning session.

The President appointed as such committee, Dr. D. C. Dewey, Prof. McGugin, and Dr. J. Siveter.

On motion, the Society adjourned to 7, P. M.

EVENING SESSION—7, P. M.—The Society met pursuant to adjournment, and was called to order by the President. The minutes of the afternoon session were read and approved.

On motion, the Society proceeded to the appointment of delegates to the American Medical Association.

Drs. Rauch, G. R. Henry, and M. Cousins, were appointed delegates; Drs. Wood, Adamson, and McGugin, alternates.

In the absence of Prof. J. R. Allen, who had been appointed to deliver the annual address, Prof. McGugin, upon request, delivered an extemporaneous address, after which the Society adjourned to the ensuing day at 9, A. M.

MORNING SESSION.—Society met pursuant to adjournment, and was called to order by the President.

The minutes of the ensuing session were read and approved.

The Constitution and By-Laws were read, and the Committee appointed to revise the same, then made their report.

The report of the Committee was accepted, and the Committee discharged.

The report was then taken up, and after discussion and amendment, was adopted as the Constitution and By-Laws of the Society.

Dr. Kinsey then offered the following preamble and resolution, which were adopted:

WHEREAS, The Medical Department of the Iowa University is in the highest state of prosperity, and by its efficiency in the elevation of the standard of the profession in the State, has secured the almost undivided confidence of the profession generally, and more especially that of the State; therefore it is

*Resolved*, That we most earnestly recommend this Institution to the continued favor and confidence of the profession, and to the educational authorities of the State, as worthy of their maintenance and constant protection; and as it has been eminently successful in the accomplishment of its objects, we would express the opinion that State pride, as well as considerations of humanity, should demand from the people, the most zealous efforts for its continued support.

The Committee appointed to memorialize the Legislature was continued—Professor McGugin, Chairman.

The Committees on Anatomy, Physiology, Obstetrics, and Practice, failed to report.

Professor Hughes, of the Committee on Surgery, made his report, after which, on motion, the Society adjourned to 1, P. M.

AFTERNOON SESSION.—Society met pursuant to adjournment, and was called to order by the President.

Drs. W. B. Cochran and J. C. Stone presented certificates of election as delegates from the Iowa City Medical Association, and Dr. J. C. Evans the same from the Davis County Medical Association. These gentlemen, on complying with the requirements of the Constitution, became members of the Society.

Drs. Stone, Fitch, and Thrall, were appointed a committee on Ethics.

Prof. McGugin made a verbal report of an interesting case of ossification of the œortic valves, presenting the morbid specimen of the same.

On motion of Dr. Wood, it was resolved that a synopsis of the proceedings of the Society be published in the Ottumwa papers.

The President appointed Drs. Woood and Cousins a committee to make the synopsis.

On motion of Dr. Rauch, it was resolved, that the transactions ot the Society, for this year and the last, with the Constitution and By-Laws, be published in pamphlet form.

Drs. Dewey, Cousins, McGugin, and Wood, were appointed a publishing committee.

On motion of Dr. Rauch, it was resolved, that a committee of three be appointed to memorialize the Legislature, in relation to an enactment directing a registration of births and deaths throughout the State.

The President appointed Drs. Rauch, Cochran, and Marsh, such committee.

On motion, it was resolved, that the thanks of the Society be tendered to the profession of this place, for the kind attentions which have been extended to its members during its session here..

On motion, it was resolved, that the next annual meeting of the Society be held at Iowa City.

Dr. Cochran was then appointed to deliver the dissertation prescribed by article 1st of the By-Laws, at the next annual meeting.

The President appointed the following committees, to wit:

*Anatomy.*—Dr. Siveter, Salem; Dr. Fountain, Davenport; Dr. Page, Keokuk.

*Physiology.*—Dr. Dewey, Keokuk; Dr. Cleaver, Columbus City; Dr. Huey, Fairfield.

*Therapeutics.*—Dr. Marsh, Keokuk; Dr. Fitch, Chariton; Dr. Taylor, Ottumwa.

*Pathology and Practice.*—Dr. McGugin, Keokuk; Dr. Cochran, Iowa City; Dr. Robertson.

*Botany.*—Dr. Cousins, Albia; Dr. Rauch, Burlington; Dr. Y. Collins, Chariton.

*Surgery.*—Dr. Wood, Ottumwa; Dr. Siveter, Salem; Dr. Reeder, Muscatine.

*Pulmonary Consumption.*—Dr. Stone, Iowa City; Dr. Adamson, Nevada; Dr. Hinsey, Dahlonega.

*Obstetrics.*—Dr. Allen, Keokuk; Dr. Elbert, Van Buren county; Dr. Bird, Mt. Pleasant.

*Quackery in and out of the Profession.*—Dr. Rauch, Burlington; Dr. Gauch; Dr. Knowles, Keokuk.

On motion, it was resolved, that the publishing committee be empowered to draw upon the Treasurer for moneys to defray the expenses of publication.

REVISED CONSTITUTION AND BY-LAWS OF THE IOWA STATE MEDICAL SOCIETY,

Adopted at its Annual Meeting, June, 1856,

For the purpose of harmonizing the profession of Medicine, and of promoting its usefulness and respectability, the undersigned, practitioners of Medicine, in the State of Iowa, do adopt the following Constitution, to wit:

ART. 1.—This association shall be known as the Iowa State Medical Society, and shall hold its regular meetings on the second Wednesday of June of each year, at such place as the Society, at its next previous meeting, shall have determined.

ART. 2.—The officers of this Society shall consist of a President, Vice President, Recording and Corresponding Secretaries, Treasurer, Librarian and seven Censors, and shall be elected annually, the President by ballot, and the remaining officers in such manner as the Society may direct, a majority electing.

ART. 3.—It shall be the duty of the President to deliver an address before the Society at each annual meeting, to preside at all meetings of the Society, to give a casting vote except at elections, and to perform all other duties consistent with ordinary parliamentary rules.

ART. 4.—It shall be the duty of the Recording Secretary to keep a record of all the proceedings of the Society, and report at each annual meeting. It shall also be his duty to issue a certificate of membership, to each member desiring the same.

ART. 5.—The duties of the Corresponding Secretary shall, in all cases, be performed under the direction of the Society.

ART. 6.—It shall be the duty of the Treasurer to receive and have charge of all moneys belonging or accruing to the Society, to disburse them according to its order, and to hand over any in his hands to his successor in office. He shall also report at each annual meeting to the Society.

ART. 7.—It shall be the duty of the Librarian to take charge or any books or specimens belonging to the Society, and keep the same under such regulations as may hereafter be ordered.

ART. 8.—Any regular Practitioner, in good standing, may become a member of this Society by a vote of a majority of the members present at any annual meeting. But it is provided that his name shall first be presented to the Society by a member, and he shall also exhibit to the Society a diploma from some respectable Medical College, or a license from some respectable Medical Society, and shall pay an initiation fee of two dollars.

ART. 9.—The code of medical ethics adopted by the American Medical Association, is also adopted by this Society, and any member who shall violate this code shall be liable to expulsion from the Society, by a vote of three-fourths of the members present at any regular meeting, or to censure.

ART. 10.—This Society, by a vote of the majority of the members present at any annual meeting, shall have power to levy a contribution upon its members to meet any necessary expenditure.

ART. 11.—Any article of this Constitution may be amended or abrogated at the annual meeting in June, provided three-fourths of the members present concur therein.

### BY-LAWS.

ART. 1.—This Society shall appoint at least one person at each annual meeting, to deliver a dissertation at the next annual meeting, and if such person be not present, the Secretary shall advise him of his appointment.

ART. 2.—It shall be the privilege of any member of this Society to report, at the regular meeting thereof, any important cases that may have come under his observation.

ART. 3.—The order of business, at the regular meetings of this Society, shall be the same, as far as practicable, as that observed at the meetings of the American Medical Association.

ART. 4.—The Secretary shall give four weeks notice, in at least four of the public papers of the State, of the time and place of each annual meeting.

ART. 5.—It shall be the privilege of the President and Recording Secretary, upon the application of five members of the Society, at any time, to call an extra meeting, for the purpose of considering such business as may be necessary to be brought before the Society, previous to the succeeding annual meeting; and it shall be the duty of the Secretary to give suitable public notice of such extra meeting.

ART. 6.—A committee of three shall be appointed at each annual meeting, to whom all complaints of violation of the code of ethics shall be referred, and no action shall be taken on any complaint by the Society until reported upon by the committee.

ART. 7.—Three members of the Board of Censors shall constitute a quorum for the transaction of any business coming under their direction.

### REPORTS OF CASES.

### REPORT OF A CASE BY THOMAS SIVETER, M. D.

On the 13th of the month, in the night, I was called to visit P. L. Dr. S. was already in attendance, and upon my arrival, engaged in the attempt to introduce the catheter. P. L. had, previous to the arrival of Dr. S., suffered violent pain, and had introduced the catheter himself, as he had been in the habit of using it. Instead of urine passing, a profuse hemorrhage occurred. I suggested that P. L. had not introduced the catheter into the bladder, but had produced a false passage, which was the cause of the hemorrhage. They (Dr. S. and P. L) said no! for Dr. S. had by use of catheter evacuated from ℥iij to ℥iv of urine. I was by this information puzzled.

I proposed withdrawing the catheter and introducing a larger one—say No. 10—my faith still being alive with regard to the false passage. On withdrawing the catheter it was filled with coagula. The No. 10 catheter was introduced. No urine passed, but blood was evacuated. We attached an elastic bottle to the catheter and injected tepid water, but it returned by the urethra, outside the catheter. As I was beaten out of the false passage idea, I thought it possible the bladder might be distended with coagula, and so we adopted that notion. Before removing the catheter, we injected an aqueous solution of opium, but it returned the same way as the water. On the removal of the catheter, it was again filled with coagula, as before.

Large doses of opium, with acetate of lead, were ordered, together with the warm bath. The warm bath rendered relief from pain, but not permanently.

Upon examination of the rectum I found enlargement of the prostate.

I then left Dr. S. in charge of the case. In the after part of the next day I called in again. No relief; no urine passed. Warm bath from time to time. Opium and Acetate of Lead had been used, and the catheter repeatedly introduced, and still the hemorrhage continued. Dr. S. and P. L. said that I must now take the case and do the best I could with it. (Rather a sorry bequest.) The coagula bugaboo still in the ascendant. Continued the warm bath, (nothing else relieved the agony of the patient.) Ordered Opium and Gallic Acid, and quit using catheter until return visit next morning.

Morning: Patient expressed himself as much better. No hemorrhage; had passed some urine involuntarily, and wet the bed; was quite easy. I considered him to be much worse; bladder was enormously distended, reaching almost to umbilicus; fæcal matter in rectum, flattened out to a line or so in thickness, and the outline of enlarged prostate well defined. From the attenuated condition of the coats of the bladder, I felt confident my first diognosis was the correct one.

Called Dr. S. in again and proposed puncturing the bladder per rectum, but concluded first to make a very careful thorough attempt with the catheter. Happily we succeeded, and a very large chamber-pot more than half full of urine was evacuated.

I may here state that I had left the introduction of the catheter from first to last to Dr. S. and the patient, in no instance having myself tried it, and considering all the circumstances, do not feel myself very culpable for the coagula difficulty.

Well, every thing was much better, but paralysis of the bladder was the natural sequence of the previous over-distention.

We left the catheter in the bladder, secured to prevent its slippping out, but in six or eight hours it was, nevertheless, expelled upon the occurrence of a fæcal evacuation.

It was afterward introduced from time to time until the patient was taught to introduce it properly.

Remember, by this time it is the 31st of the month. P. L. has an inquinal hernia of the left side; has worn a truss until laid up by his late exigency, during which time it was left off.

Convalescing, he resumed his truss, rode out, walked about in the garden, &c., but two days since consulted me about some œdema of his left leg and thigh. There was no pain, but some stiffness along the course of the femoral vessels, and in popliteal space. Told him to lay off his truss, and assume recumbent position, which would probably tend to reduce the swelling. To-day (31st July) sent for me; found the œdema much increased; more stiffness; and, on close questioning, the patient says that when he first resumed the truss it hurt him very much. I now discover much tenderness deep in the iliac fossa. Put him on the use of calomel and opium, and directed tepid fomentations to iliac region. To retain recumbent position, and to use frictions on the limb, the pressure to be directed from distal extremity to center.

This phlebetis make the case interesting. The source from which it emenates, and what its results, are matters of question; but I will note the course of the case in as brief a manner as is consistent with perspicuity, as it progresses.

Eighth month—first day—morning. Less œdema; not so much stiffness; continued fomentations; gave calomel and opium, but diminished the dose to one-half—that is, calomel, gr. j; opium, gr. ¼—every four hours. No new developments or anything more worth further notice.

2d. Œdema still lessening; less tenderness on pressure in iliac fossa, but a spot say four inches below Poupart's ligament, and in the track of femoral vessels, quite tender. Continued calomel and opium; applied warm stupes over tender space, and continued to use frictions, as before. The tongue is to-day brown and dry; pulse accelerated and hard; no appetite. I should have remarked that the pulse was slightly intermittent, (once a minute perhaps,) but patient says it is so in his best state of health.

3d. No visit.

4th. Gums tender; oedema still diminishing; had four or five evacuations from bowels during the night, with considerable pain about sigmoid flexure of colon during the passage of the fæces. Pulse and tongue about the same as at last visit; omitted calomel and opium; washed the mouth frequently with infusion of peach leaves.

5th. Œdema still less; some moisture of the tongue; several stools during the night with less pain, and one this morning without any pain; sweat profusely during the night, and has done so for several nights past; appetite poor; pulse considerably softer; more heat in swelled leg and thigh than in the sound one. The catheter, as he expressed it, passed by its own weight quite easily; introduc has to it every six or eight hours.

7th. Much better; tongue cleared off; appetite good; gums less tender; pulse nearly normal; both limbs are of same temperature, and œdema nearly gone; there was some stiffness along the sartorius and recti muscles; he is sitting up, and I consider him convalescent.

I have not time to hunt up precedents in case of Phlegmatia Dolens, but have the impression that everything is done and recommended by the text books, but what is most effectual, to wit: mercurialization. Dr. Graves, of Dublin, in his clinical lectures, recommends the mercurial plan. I have had several cases latterly treated as this one, and with the like happy result.

REPORT OF A CASE BY D. L. M'GUGIN, M. D.

I was requested, in April last, to visit an old lady, aged 70, who, it was said, had been laboring under dyspnœa, with cough, suffocation, palpitation of the heart, loss of appetite and great distress.

Pulse 100, feeble and intermitting, emaciation, great jactication, inability to lie in any other position than a recumbent one, a livid circle about the eyes, lividity of the lips, skin cool with a very feeble capillary circulation, hurried respiration, rational but irritable, uneasy and restless, were the symptoms which first arrested my attention.

Physical exploration of the chest exhibited dulness upon percussion over the chest, but in auscultation the bronchial respiration was so loud as to obscure wholly the vesicular murmur if the air did reach the ultimate cells. The heart's action was irregular, sometimes ceasing altogether its regular contractions, then recurring again in paroxismal efforts to send out the blood. The distinction between the first and second sounds was wholly lost and indeed the whole act was performed as with a sudden and powerful struggle. I detected a kind of hissing sound, indicating a great lessening of the aperture at the mouth of the aorta or near it. During the struggles of the heart, the face would become livid, the body would be elevated to a sitting posture to permit her to breathe, and the distress attending the effort would be very great. There was also great pain in the region of the heart. These symptoms recurred so frequently and the restlessness was so great, that the examination was necessarily attended with much difficulty and could not be persisted in.

I was therefore unable to determine, with any approximation to ordinary accuracy, the pathological condition of the heart, but believed at the time that there was an impediment to the free passage of the blood from the heart and suspected the existence of a tumor compressing upon the aorta at its origin.

I requested to be notified when death took place, as I desired an autopsy if there were no objections by the family, in relation to which a consultation might be had by them and their friends. In the morning I was advised that she had died during the night, and that no objections would be made to a post mortem. Aided by several medical friends, I proceeded to an examination.

The lungs were melanotic throughout and much atrophied. The bronchia dilated, the liver was livid, hyperthropied and in a condition of toughness equal to gum elastic. In the chest was a large quantity of serum, and the pleura costalis highly congested. The heart lay with its apex over to the right of the line, and the main body in the centre was much hyperthropied, with a large amount of serum in the pericardial sac.

But no tumor or other cause of impediment yet was found. The heart was removed. The auricles were distended, particularly the right, which was very much thinned in its walls. The right ventricle was also enlarged in its cavity, and also thickened in its structure, but the left ventricle was still more enlarged, its walls three-fourths of an inch thick, and there was a clot in each cavity. Following up the examination, I found that instead of the aortic valves, the mouth of that vessel was closed by an ossified septum, as with a narrow fissure through the centre, from one side to the other, thereby dividing the ossified portions into two equal sections, or hemispheres, but so narrow and close as to permit only the introduction of a thin knife blade.

The peculiar whizzing or hissing sound was now accounted for, and its clear intensity, if I may be allowed the expression, readily explained. To force a column of blood, deprived of much of its watery constituent, through so narrow an apperture, would not only require a great effort on the part of the contractile fibres of the heart, but as the edges of this fissure was of unyielding material, deriving its mobility only from the soft structures to which it was attached, the sound emitted would obviously be more intense and striking, and much more so than when passing through a similar sized apperture in soft and yielding structures. The hyperthropy of the heart to double its natural size could also be accounted for upon the ground that constant exercise and the continued demand for great effort had contributed to it. But the great thinning of the walls of the right auricle may be more difficult of explanation. This I account for on the ground that the great distinction was not reciprocated by those circumstances and agents which contributed to the increase in thickness of the wall of the ventricles, for but little force is exerted by the muscular fibres of the auricles, inasmuch as gravitation does away, to a great degree, with the necessity. Nor were the walls of the right ventricle thickened in the same proportion beyond their normal state as the left, for the same reason, there not being the same power required on account of the shorter distance. And yet the force *was* increased beyond the normal requirement, and hence, too, the abnormal thickening. We cannot overlook the part which the lungs sustained in this general diseased condition of the thoracic organs. The blood came to the right auricle demanding admission—here it accumulated, awaiting the movement of the right auricle; this last had the duty of forcing the blood through the pulmonary artery, in which office it was embarrassed, because of the great engorgement in the pulmonary vessels. Even suppose it competent to the discharge of its duty, it must act synchronously with the left ventricle, which, if embarrassed from any cause, its action might be delayed, which would also delay the movement of the action of the right. In this case, then, we would have engorgement of the right auricle and of the ventricle of the right heart, with regurgitation back into the veins, perhaps.

But the blood passing through the lungs seek admission into the left auricle, but there is a hesitancy to its admission. This would take place from the unyielding barrier set up at the aortic orifice. The blood could not pass freely on its round in the systemic circulation, and being arrested in the left ventricle, and while this cavity was filled, of course the auricle of that side could not relieve itself, nor could the pulmonary vein unload itself, the venous capillaries would therefore become engorged by regurgitation, the arterial ramifications would be over-burthened because not discharged, and the artery would be overloaded with venous blood, appealing for oxygenation and life, while the right ventricle must allow itself to distend to permit the ingress of the inpouring tide. Here we find auricles, ventricles, vein, capillaries, and artery engorged and distended.

The melanosis of the lungs, if we reject Andral's views of malignant growth, can be readily accounted for upon the ground of deposition. This melanoic condition which presented different aspects, embraced the entire lobes of both lungs, sometimes solid, then rather semi-fluid, but giving to the surface of the lung a tuberculated appearance, the bodies appearing intensely black. Some of them appeared encysted and the contents of more or less consistence, but generally they were fluid. If, as is supposed, it is an altered coloring matter of the blood, this was a case which would give force to the conclusion and good reason for its adoption. The blood arrested here in the lung, without the opportunity of decarbonization, would necessarily become depraved, and deposition and infiltration followed the great engorgement of the vessels. These depositions had doubtless been forming from time to time, but recently more actively because of the greater proportionate number of the semi-flluid bodies. Then we had in this case, first, congestion; second, imperfect oxygenation; and then, third, infiltration and deposition, aggravated and increased by the increasing congestion, of a highly carbonaceous material resembling black pigment. In this way we account for this peculiar and rare pathological condition of the lungs.

The effusion within the cavity of the thorax would be looked to by any one who had ever studied the pathology of effusions in connection with organic changes of the heart.

The condition of the liver, in particular, and other secreting organs, would also be looked for. Hepatic congestion would be looked for as an attendant upon the conditions above described. The imperfect respiration would illy qualify the blood and therefore it was highly carbonized, which was shown by the cyanotic aspects of the face and extremities.

The obstruction to the free return of the blood from the brain would favor engorgement of the sinuses, the compression of which would superinduce a comatose condition. Besides this, the brain would be poisoned by impure blood, and, under these circumstances, asphyxia would, as it did, result.

I have thus gone somewhat in detail in this case, because it is another instance, among many others on record, in which a cause, "trifling in itself," has produced a series of pathological changes, resulting seriously, because not under control.

REPORT OF THE COMMITTEE ON SURGERY, BY PROF. J. C. HUGHES, M. D.

As chairman of the committee on Surgery, I beg leave to submit to your honorable body the following report:

In all new States and countries, it is expected that less attention will be devoted to the cultivation of science and art than where, as in older states, society is established, institutions of learning in the highest state of efficiency, and where wealth is possessed to make possible the pursuit of knowledge under the favoring influences of these benefits and blessings.

When ability and opportunity combine to make the attainment of intellectual advancement easy to all, it is hardly a virtue in those who embrace the advantages thus afforded that they do so. Nor is it strange, that, under such propitious circumstances, some attain to superior accomplishment in those departments towhich they devote their attention.

In Europe and the older portions of our own country, advantages are possessed for acquiring knowledge in all departments of scientific and literary learning, which we in the west may not, for a long time, enjoy. Libraries and cabinets, such as old institutions and men of wealth can alone be supposed able to possess, and which may be acquired only with long continued efforts, and the slow accumulation of years, are theirs, and are important agents in the dissemination of knowledge and in scientific culture.

But in the west, these advantages are yet to be acquired. True, in this country the march of improvement is rapid, is indeed a rushing whirlwind—onward, lengthening, strengthening, deepening like a mighty river, as it advances, until its tide and civilization has already reached beyond the Rocky Mountains—making the rude hamlet of yesterday, the city of to-day—the wilderness of to-day to blush as the rose to-morrow—rearing, with magic celerity, the temples of God and of science upon the spot whose earth is yet warm with the fires of the infidel and the savage.

But the bustle of pioneer life does not dispose to arduous study, is poorly adapted to the encouragement of quiet research and patient investigation, so necessary to a high grade of literary and scientific accomplishments. The sound of hammer and axe—the driving of the plough and wielding of spades—the excitement of speculation, and the various industrial pursuits to which we are driven by pecuniary necessities, very naturally consume our time and energies and banish, to a great extent, more congenial pursuits.

Professional men, and particularly those of our own profession,—poorly remunerated for their services, are compelled to devote the time of relaxation from professional duties to purposes which will yield a pecuniary reward, that they may thereby have the necessary comforts for themselves and families.

Under such circumstances it is a matter of surprise, not that so little, but that so much is accomplished in medicine and surgery to elevate and perfect these sciences by the profession in the west.

In respect to Surgery, to which it is expected your committee will confine their report, it is with a feeling of pride that they are enabled to congratulate you in view of the position which many of the surgeons of Iowa have carved out for themselves, a reputation notwithstanding all the circumstances of an adverse character by which they are surrounded; unable, because of the comparative sparsity of our population and the limited number of cases which a robust and healthy community, mostly devoted to agricultural pursuits, present for surgical interference, and the fact, too, that the surgeon, with us, is compelled to perform the duties of general practitioner of medicine, unable, from these causes, to acquire the mechanically practiced hand of the surgeons of our large eastern cities, yet they have proved themselves fully masters of the principles of the art.

Nor has the ability to operate promptly and successfully been found wanting, when such assistance has been demanded, although in some cases perhaps the refinements of invention in instruments, and some of the extreme niceties in manipulation may be wanting.

The habits of life in a new country, however, have the effect of begetting in all classes a feeling that to him who *wills* there is a *way*—that, next to a correct understanding of principles, a well grounded self-reliance is necessary to success. This, together with the fact that familiar consultations with medical brethren are oft times impossible, constitutes a very natural cause for what we deem to be true of western Surgery, viz: that their action is ever prompt, and their recourse to medical measures characterised by a boldness and independence rarely witnessed elsewhere by those of equal practical experience. And yet the record will compare favorably, as respects success. But while inclined, in view of the character of the Surgeons of Iowa, to speak with some show of arrogance, your committee would not wish to be understood as being disposed to disparage or detract from the merits of critical study, acute investigation, refinements in invention in Surgical appliances, the usefulness of practical skill nor an educated hand in the practice and operations of Surgery. Nor, while extolling the Surgery of our young State and the character of those engaged in its practical duties, do we consider that we are so doing.

Success in Medicine and Surgery, to be constant must be deserved; and were we unable to speak favorably of the science and skill of our Surgeons, from actual knowledge, we should be convinced by reasoning that as their success is almost constant, they do deserve it, which would be inconsistent with all experience, were they ignorant of the principles of what they practice.

But it is also to be remembered that in our young State the Profession is adorned by many who had won for themselves an enviable reputation in Medicine and Surgery previous to immigration and settlement among us. We have cause for pride in showing that while the country swarms with empirics of every grade—while empiricism lifts its front boldly and unblushingly as sin itself, and feats and flourishes, growing fat and proud, that the legitimate profession have taken a position for respectability and true worth among the better and more intelligent portion of community which the followers of the various medical *isms* may ape but never attain to.

These rarely attempt Surgery, as fortunately its results are palpable and capable, in a degree of apreciation by all.

But again, your Committee are happy to be able to state, that to a considerable extent there is a manifest assiduity among the profession of the State in the pursuit of those sciences upon which the principles of the Surgical art must ever rest, and to a thorough knowledge of which it must ever look for practical success.

Anatomical and Pathological investigations, notwithstanding the difficulties which the state of society offer to their prosecution are not premitted to fall into entire neglect. It is true that for reasons already enumerated there is too great apathy respecting these important branches of medical and surgical science among the profession generally but there are praiseworthy exceptions, not one or two only, but a considerable number of them, to this rule.

Cabinets—private and public, are being made up which though small now, yet from these substantial nuclei of what in time will become large and valuable collections and do good service, it is to be hoped, in awakening the profession generally to the importance of the sciences they are intended to illustrate. It is only by such means, by the cultivation of these sciences, that surgery here or elsewhere shall become such in the true and higher sense of the term—a surgery which is able not only to determine how but *when* to operate and when to let alone.

Iowa is young but it has its future, and it needs no prophets vision to reveal what that future is to be; so, too, its surgery is young but it also has a future and what that shall be is to be decided by those into whose hands it has fallen. We feel that it is now occupying a creditable position for so new a country, but not such as it yet must occupy. The obstacle in the way of advancement are daily growing less and there is no room for doubt that those who have not been deterred by greater obstacles in the past from laboring for its elevation will now do it with increased honor while those who are entering, or are hereafter to enter upon its active duties, will be stimulated to effective zeal in the endeavor to give to the character of Surgery and Surgeons in Iowa a reputation which shall not suffer by comparison with that of the older States.

Such, as we have expressed it, are the views of your committee upon the present condition and future prospects of Surgery in

Iowa; but we cannot close without asking of your honorble body their indulgence for the general manner in which we have treated the subject.

We had intended to have gathered some statistics of what had been accomplished in the State, in this department, since the last report of the committee to this body. This we have been unable to do so as to make such report anything like a full statement or to place the matter in a proper light before the profession.

The principal cases which we are able at this time to present have been taken from my own practice and of these the most important are those of calculi in the bladder of which six cases have occurred in my practice since I last met with you.

I shall briefly refer to the different cases, not having been able to complete a full report.

CASE 1st. Mr. Short, aged about 40, of Henry Co., Iowa.

This case was fully reported in No. 3, Vol. 2. of the Iowa Medical Journal, and I shall but briefly relate to it in this report.

The case was one of particular interest, from having been unsuccessfully operated upon by the lateral method, before consulting me. In this operation the rectum had been wounded, and adhesion between it and the bladder followed, rendering the second operation more than ordinarily difficult, and calling for particular nicety of manipulation.

I chose the bi-lateral method, and performed it with entire success, removing a calculus of the triple phosphate variety, measuring one inch in length, two-thirds in width, and two and one-quarter inches in circumference: weight over five drachms.

Recovery was rapid,—within thirty-six hours after the operation the patient passed nearly all the urine through the urethra, and on the seventh day the wound was so far closed that all passed by the natural channel.

On the tenth day the patient was walking about, and on the twelfth day returned home—some fifty miles.

CASE 2nd. Son of Mr Tresler of Keokuk Co., aged 6 years.

In November last Mr. T. presented his son for examination, stating that for two or three years he had occasionally suffered symptoms similar to those then presenting themselves,—that for the preceding two or three months, his sufferings were excruciating. Stone being plainly indicated, the sound was passed and the diagnosis confirmed.

I operated upon the little fellow before the medical class of the University, November 7th., 1855, after the bi-lateral method with the double lithatomie cache. This stone was also of the triple phosphate variety, weighing over half an ounce. Twenty-four hours after operation most of the urine passed, and on the third day all the urine passed by the natural channel. On the ninth day the little patient was playing about the floor, and on the tenth left for home, entirely well.

Case 3d. Son of Mr. Penwell of Farmington, Van Buren connty, Iowa, a very interesting little fellow, four years of age, who had been an occasional sufferer, for some two or three years. The extent of the suffering was much less than in the second case, and the symptoms not so plainly indicative of calculus. The history given of the case by his parents led me to diagnose stone, and the introduction of the sound confirmed the same.

I first saw the patient at Farmington, in the early part of November, and on the 26th of the same month the operation, (bilateral,) was performed before the medical class.

The success in this was fully equal to that in the last case, and the patient was able, on the tenth day, to leave for home. The calculus, in this instance, was of the mulberry or oxalate of lime variety, and about the size and form of an ordinary almond kernel.

Case 4th. Mr. Hier, aged 32, of Van Buren Co., Iowa.

This man presented himself for treatment in November last, and stated that his physician, Dr. Smith, of Farmington, had pronounced his case to be one of Gravel, and advised him to place himself under my care; and, upon inquiry, I learned he had been a sufferer from similar symptoms to those now presenting themselves for nearly eight years. He was much prostrated with hectic symptoms and general anæmia, having been confined to his bed for nearly two months. There was a copious, unhealthy muco, purulent discharge from the urethra, and abscess of the penis communicating at one part near the bulb with the urethra. I introduced the sound, and, without difficulty, detected a calculus of considerable size; also, found excessive thickening with contraction of the walls of the bladder. His condition would not justify an operation at the time, and he was immediately sent to the hospital and put upon a treatment with a view to improving his condition sufficiently to warrant an operation for his permanent relief, but without any noteworthy beneficial effect. He continued to decline rapidly, and in some three weeks died.

The body was brought before the medical class and operated upon by the lateral method, removing two calculi of large size, of the mulberry or ovalite of lime variety. The dissection was afterward continued, revealing great thickening and contraction of the walls of the bladder, with softening and abscess connected with its lining membrane. One of the kidneys was in a healthy condition, yet much hypertrophied; the other was nearly destroyed by suppuration, indeed had been converted into a purulent sac—the medullary portion was broken down—the cortical substance only remaining to form the sac for a mass of unhealthy pus.

Case 5th. Gen. Whitmore, age 62, of Jefferson Co., Iowa. This gentleman was one of the early settlers of Iowa, and one of the most active and energetic citizens of the State, although an occasional sufferer from this affection for the last twenty years. In February last he requested me to visit him, giving me an intelligent description of his case, stating that his sufferings were very great and he was satisfied that nothing could relieve him except an operation. He wished me to come prepared to operate. I visited him on the 8th and found him considerably prostrated, with thickening of the wall of the bladder and a profuse muco purulent discharge from the urethra. He was conscious of his danger, either with or without an operation, and preferred the latter. I proceeded to operate by the bilateral mode—removing two stone of the triple phosphate variety, the largest about the size of a large hazle-nut and the other about one-third the size. The result in this case was favorable, though not so rapid as in the former cases, owing, no doubt, to the condition of the parts as well as the age of the patient.

Case 6. Mangus Hanson, age 64, Burlington, Iowa. This old gentleman had been a sufferer for nearly thirty years, but owing to his sedentary habits, he was, until within the last two years, a stranger to the more aggravated form of the disease. On the 17th of May last, at the urgent request of Dr. Day, of that city, who was his attending physician, I visited him. The Doctor had previously sounded him and detected stone. It was therefore only necessary to decide as to the propriety of an operation.

The patient was examined; considerable thickening of the bladder as well as a discharge of unhealthy muco purulent matter. The case was not in good condition for operation; but taking everything into consideration, we decided in favor of the operation, and I proceeded to operate by the bilateral method, removing a stone of about half an ounce weight, of the triple phosphate variety.

This case had a fatal termination, but the annexed letters from Dr. Day would seem to warrant the conclusion that the death was attributable to cause other than the operation.

Burlington, July 22, 1856.

Prof. Hughes—Dear Sir:—I write you a few lines to-day in regard to our patient.

Hanson is doing as well as we could probably expect. His urine came *per via naturalis* on Sunday morning, but no more until to-day (Thursday). He has not much pain and not quite as much inflammatory action as I could wish. I gave him a cathartic Tuesday, and shall give him another to-day.

He is anæmic, and to that I attribute, somewhat, his tardy reaction, and I shall treat him accordingly. He suffers considerable pain when the urine passes per urethra, for which I prescribed the preparation of Copaiba and Benzoic acid.

He will probably recover, very slowly, but, I think, surely.

Yours, in haste,

W. T. Day.

BURLINGTON, June 6th, 1856.

*Prof. Hughes*—Dear sir—I have neglected to advise you of the course of affairs here, with our patient, and can only plead neglect.

He showed no signs of re-action after I last wrote you and only passed water, per urethra, on the eighth and ninth days. He seemed to suffer great pain in the region of the epigastrium, although I could discover no particular lesion of the stomach. He had a tenderness of the abdomen and a greenish, curdy, discharge of a very fœtid character. He gradually wasted, having no appetite and no assimilation of what he forced down, and became so weak as to be unable to turn in bed.

Yesterday he died and on a post mortum, this morning, with Dr. Harvy, we found the whole thing evident. To one seeing the kidneys, there would only be a wonder of his existence so long. There was a complete granular degeneration of both, and the substance mostly broken up into large abscesses, containing pus of a very fœtid character. I should say there was, in the two kidneys, not less than eight ounces of this. There was hardly a trace of the kidney proper left—the left double size, the right one and a half times natural size; the bowels, particularly the illeum, somewhat injected.

The bladder was a thickened mass, secreting pus from its entire inner surface. I should have said that no urine was secreted or passed for forty-eight hours before death.

I have one of the kindeys here and, if you wish it, I will send it to you, as it is a very interesting pathalogical specimen.

Yours,

W. T. DAY.

P. S.—I have just weighed the kidney, which, instead of Wilson's three to five ounces, weighs eight and one half ounces heavy. W. T. D.

The calculi in the cases described were by occular examination in cases 1st, 2d, 5th and 6th of the triple phosphate variety, and in cases 3d and 4th, of the mulberry or oxalate of lime variety. Upon chemical examination it is quite probable that some two of them will prove to be compound. I have placed the different specimens in the hands of Prof. Marsh for chemical analysis, the result of which will be duly made known.

The fact of so many cases of the disease occurring within the State in so short a time, becomes a matter of interest, since it would seem to indicate that our State, or parts of it, may be, as is true of some other particular districts of country, peculiarly obnoxious to calculus diseases. It is my intention to follow up some investigations already commenced, having connection with this question, and I take this public opportunity of asking the profession generally to communicate anything occurring in their experience in practice, which they may consider of value as respects facts or opinions.

Some very interesting Surgical cases occurred in the practice of my friend, Dr. Siveter, of Salem, within the last year. But not having a report of them, I am unable to present them at this time.

Dr. Wood has related to me a very interesting case of exostosis of the inferior maxilary of a child, when it became necessary for him to remove a great part of the bone. The operation was entirely successful, and the Doctor has promised me a full report of the case.

Before closing, I would simply refer to the following cases, having occurred in my own practice.

**AMPUTATIONS.**

| | |
|---|---|
| Of the Thigh, | 2 |
| " Leg, | 2 |
| " Arm, | 1 |
| " Fore-arm, | 1 |
| " Fingers, | 15 |
| " Metacarpals, | 4 |
| Removal of Tonsils, | 4 |
| Strabismus, | 2 |
| Imperforate anus, | 1 |
| Fistula in ano, | 2 |
| Simple Hair-lip, | 1 |
| Complicated Hair-lip, | 1 |
| Fistula Lacrymalis, | 2 |
| Obstructed Punctæ Lacrymalis,' | 2 |
| Nasal Polypi, | 2 |
| Fibrous Tumors, | 3 |
| Carcinomatous, | 2 |
| Albugo, | 1 |
| Eutropion, | 2 |
| Pterygium, | 3 |
| Cataract, | 5 |
| Staphyloma of the Cornea, | 1 |
| Trichiasis, | 2 |
| Encanthis, | 1 |
| Hydrophthalmia, | 1 |
| Adhesion of Eye-lid with Cornea, | 2 |
| Hernia, | 1 |
| Trephining, | 3 |
| False Anehylosis of Maxillary Bones, | 2 |
| Ligature of Ant. Tibial Artery, | 1 |
| " Radial " | 2 |
| " Ulnar " | 1 |

**FRACTURES.**

| | |
|---|---|
| Of the Femur, | 2 |
| " Tibia and Fibula, | 8 |
| " Radius and Ulna, | 6 |
| " Radius alone, | 2 |
| " Ulna alone, | 2 |
| " Humerus, | 2 |
| " Clavicle, | 3 |
| " Scapula, | 1 |
| " Cranium, | 5 |
| " Inf. Maxillary, | 2 |

**DISLOCATIONS.**

| | |
|---|---|
| Of the Shoulder, | 3 |
| " Elbow, | 2 |
| " Wrist, | 3 |
| " Ankle, | 2 |
| " Patella, | 1 |
| " Clavicle, | 1 |
| " Phelanges, | 4 |

J. C. Hughes, *Chairman of Committee.*

[The following letter was received from Dr. Smith, giving a very interesting case, which is submitted in connection with the preceding.]

FARMINGTON, June 9th, 1856.

*Prof. Hughes*—Dear sir—I shall only attempt to give a very short history of the case that I was telling you of last night, Miss G. If I can go to Ottumwa, will enter more into detail. Saw her for the first time about ten months ago. Is 19 years of age, was troubled with dyspepsia from 11 to 14; at that time pain in the bowels of a spasmodic character commenced and continued for a short period, also pain in the right hypochondriac which continued in a greater or less degree until the present time. Simultaneous with the commencement of this pain, the abdomen began to enlarge, and steadily increased. For the last two or three years, suppression of the catamenia. Present symptoms, (first visit,) abdominal muscles distended to their utmost capacity; ensiform cartillage at right angles with linea alba. Sclerotic tinged with bile, skin the same, tongue clean, liver greatly enlarged; the right ovary about the size of a child's head at birth, bowels costive, kidneys inactive, pulse 120 and full, tenderness on pressure over the whole abdomen. Prescribed with no better success than my predecessors. In about two weeks performed the operation of paracentesis abdominis, and delivered her of eight gallons of fluid, of the consistence of albumen, of a bilious tinge when drawn; supposed to be one-third heavier than water. A sample of which is in Prof. Marsh's possession. I could then feel through the abdominal parietes several of the mesenteric glands greatly enlarged. She improved for six weeks, skin, kidneys, bowels and liver respond to the remedies. They gradually became torpid, and the abdomen to enlarge, and now for the remarkable feature of the case: Immediately on the upper edge of the umbilicus, a small bulb made its appearance from which the fluid in the cavity of the abdomen exudes guttatim, from a quart to a half gallon in twenty-four hours. She has perfect control over the discharge—continues while on the side or sitting up, and ceases while lying on her back. I denominated it a spontaneous paracentesis abdominus. I should have mentioned, no alteration in ovarian tumor. In my hurry I have given you a very imperfect history of the case—depending on my memory. Have not mentioned the treatment, as my particular object was to call your attention to remedio naturæ. Respectfully,

JOSEPH A. SMITH.

Since the adjournment of the society, I was informed that Prof. McGugin had seen the above case in consultation, and I requested a statement from him of the history and of the termination of the case, and he reported to me as follows:

"I did not meet Dr. Smith in the case when I arrived, in consequence of an engagement on his part, which could not be neglected, but I found there my young friend and former pupil, Dr. Laws, the partner of Dr. S. As he had occasionally seen the case, his description of it, though more full, accorded with that hurriedly given by Dr. S. to you, and which you reported to the Society.

I found the patient of sanguineous temperament, anæmic, pulse small and frequent, the skin exsanguined, the tongue and gums pale, the feet somewhat œdematous. The spontaneous opening in the umbilicus still remained, through which, when the patient was placed in a favorable position, there was discharged a large amount of serum daily. The right ovary was prodigiously enlarged, but no fluctuation could be felt through the integuments. It was then examined through the vagina, but although it could be felt distinctly, no fluctuation was detected.

She was directed to take sulphate of iron in combination with antispasmodics, and to indulge in nutritious food.

Since then I learned from Dr. Smith personally, that she improved so far as to be able to ride to the neighbors; in which condition she continued for some time, but finally the pain returned in the enlarged ovary, which increased, and resulted in suppuration, ulcerating in the direction of the vagina, into which it discharged a large amount of pus from time to time, until she was exhausted and sank. Such were the facts, as they fell under my own cognizance, and such I learned from others to have been its history since I saw it. The patient resided about thirty miles distant, and therefore I heard nothing of the particulars until after she died. There was no autopsy, to the regret of all, as the time of her death was not known to the attending physicians.

### PROCEEDINGS OF THE HANCOCK MEDICAL ASSOCIATION.

CARTHAGE, ILLS., July 16th, 1856.

Hancock Medical Association met, the President in the chair. The President read an able address on the "Duties of Physicians and Patients," a copy of which was, on motion, requested for publication in the Carthage *Republican.*

Dr. Hall read a paper on the Pathology and Treatment of Acute Dysentery.

Dr. Pierson related the following facts, relative to a case of supposed intra-uterine fracture of the clavicle.

"A female fell on the edge of a board six weeks before her expected accouchment. Severe pain and other symptoms of premature labor followed, but by rest and the administration of anodynes, she was enabled to complete her term. At birth a kuckle-like prominence was observed on the left clavicle of the child, which was probably occasioned by a fracture, with displacement."

[It is said that Chaussiear dissected a fœtus that had 113 fractures. See Erichsen's Surgery, American edition, page 182.]

The doctor also stated that he had recently met with three cases, where the membranes had ruptured and the liquor amnii had escaped; in one case fifteen days, in another twenty-nine days, and in the third case, forty days before labor. All three were rapid and unusually easy labors, and in neither was any liquor amnii discharged during labor.

Dr. Ellis stated that he had met recently with a case where the membranes were expelled with the fœtus entire, and that he ruptured them after all were expelled.

Dr. Ellis was appointed to prepare an oration for the next meeting.

The President appointed the following gentlemen to prepare papers for the next meeting:

On Diarrhœa—Dr. Hooten.
" Erysipelas—Dr. Coolidge.
" Puerperal Fever—Dr. Linn.
" Typhoid Fever—Dr. King.
" Remittent Fever—Dr. Booz.
" Digestion—Dr. Neal.

On motion of Dr. Hall, it was resolved that the proceedings of this Association be regularly published in the Iowa Medical Journal, and that the members of this Association be requested to subscribe for that journal.

The Association adjourned to meet in Carthage the 3d Wednesday in October. GEO. W. HALL, Sec'y.

---

At the meeting of the Buffalo Medical Association, which convened Aug. 5th, 1856, Prof. Hamilton presented a specimen of the middle finger which had been torn off by machinery. Its peculiar feature was, that in being torn out it removed with it one of the flexor tendons to the length of about fifteen inches. On the fourth day there was slight tenderness along the track of the tendon as far as the elbow, but this immediately subsided, and no inconvenience had followed. That a long narrow wound, such as this must have produced, was closed with no inflammation, is probably owing to the entire exclusion of air. Not so much that air is an irritant, but the fact that air enters implies a want of coaptation of the parts.

Dr. Howell reported a case of solar exhaustion, occurring on the 12th of July. The patient was a little girl, aged 10 years, who had been playing in the sun during the day. When Dr. H. arrived she had been lying insensible for about an hour and a half. She was pale, pulseless, and profoundly insensible; respiration slow but not labored; and her appearance did not indicate congestion of the brain. She recovered in half to three quarters of an hour under the use of stimulants, and revulsives applied externally. Dr. H. regarded it as peculiarly a case of solar exhaustion, and not of *coup de soleil*, coupling the idea of congestion of the brain with the latter condition.(?) He also regarded it as confirmatory of the theory advanced by Prof. Hunt, in the June number of the Buffalo Medical Journal, as to humidity being the active element of causation. The 12th of July was not a very warm day, but it was exhaustive; the dew-point was very high. There was rain on the 11th and 13th, but none on the 12th. The air of the 12th was humid, and the sun obscured somewhat by haze. The condition of the patient, too, was evidently one of profound exhaustion of the nervous system, and not of cerebral congestion.

